# Glucosamine sulfate suppresses the expression of matrix metalloproteinase-3 in osteosarcoma cells in vitro

**DOI:** 10.1186/s12906-016-1315-6

**Published:** 2016-08-25

**Authors:** Florian Pohlig, Jörg Ulrich, Ulrich Lenze, Heinrich M. L. Mühlhofer, Norbert Harrasser, Christian Suren, Johannes Schauwecker, Philipp Mayer-Kuckuk, Rüdiger von Eisenhart-Rothe

**Affiliations:** Department of Orthopedic Surgery, Klinikum rechts der Isar, Technical University Munich, Ismaninger Str. 22, 81675 Munich, Germany

**Keywords:** Glucosamine sulfate, Neutraceutical, Dietary supplement, Osteosarcoma, Matrix metalloproteinase, MMP-3, MMP-9

## Abstract

**Background:**

Glucosamine, a common dietary supplement, has a possible anti-sarcoma effect. However, an understanding of the underlying mechanism of such an effect is limited. For this study we hypothesized that glucosamine suppresses the basal level of matrix metalloproteinase expression in human osteosarcoma cell lines.

**Methods:**

We examined the osteosarcoma cell lines, MG-63 and SaOS-2. Cells were exposed to 0, 10, 50 and 100 μg/ml glucosamine sulfate for 48 h and treatment toxicity was determined through measurement of cell viability and proliferation. Relative gene expression of matrix metalloproteinase (MMP)-2, -3 and -9 was quantified by real-time polymerase chain reaction. Protein levels of MMP-2 and -9 were assessed by ELISA.

**Results:**

Administration of 10, 50 or 100 μg/ml glucosamine sulfate had no effect on the cell viability of MG-63 and SaOS-2 cells. A significant reduction of MMP expression in both cell lines was observed only for MMP-3, while a decrease in MMP-9 was seen in SaOS-2 cells. The expression of MMP-2 was not significantly affected in either cell line. Protein level of MMP-3 was reduced in both cell lines upon stimulation with 10 μg/ml glucosamine sulfate whereas for MMP-9 a decrease could only be observed in SaOS-2 cells.

**Conclusion:**

In this study, we found a pronounced suppressive effect of glucosamine sulfate particularly on MMP-3 and also MMP-9 mRNA and protein levels in osteosarcoma cell lines in vitro. The data warrants further investigations into the potential anti-tumor efficacy of glucosamine sulfate in osteosarcoma.

**Electronic supplementary material:**

The online version of this article (doi:10.1186/s12906-016-1315-6) contains supplementary material, which is available to authorized users.

## Background

In a landmark preclinical study Quastel and Cantero examined the effect of glucosamine on tumor growth in vivo [1]. They observed that daily administration of glucosamine reduced tumor growth and prolonged overall survival in mice bearing sarcoma 37 tumors [1]. Importantly, no toxicity was associated with the glucosamine treatment. More recently, extended clinical trials in more common tumor types underscored the potential anti-tumor effect of glucosamine in patients [2]. For example, the prospective VITamins And Lifestyle (VITAL) study showed that dietary glucosamine supplementation of glucosamine reduces the incidence of colorectal and lung cancer by more than 25 %, resulting in a 13 % reduction in cancer mortality [3, 4].

Despite the evidence for a possible anti-sarcoma effect of nutritional glucosamine supplementation, our understanding of the underlying mechanisms is limited. Early studies by Molnar et al. were restricted to describing the morphological changes that occur in Sarcoma 180 cells, a transplantable mouse sarcoma isolated from ascites [5, 6]. More recent work by Gervasi and co-workers, has investigated the effect of glucosamine supplementation in human HT1080 fibrosarcoma cells. They found that treatment with N-acetyl-D-glucosamine reduces concanavalin A-induced matrix metalloproteinase (MMP)-2 activity [7]. These findings have been corroborated by two studies conducted in Kim’s laboratory; these studies show that an amino derivative of glucosamine reduces phorbol 12-myristate 13-acetate (PMA)-induced upregulation of MMP-2 and MMP-9 in HT-1080 cells was reduced in the presence of an amino derivative of glucosamine [8, 9]. They also demonstrated that glucosamine derivatives suppress MMP-2 and -9 gene expression in addition to decreasing the proteolytic processing of MMP precursors [8, 9]. Further, work by Lin et al. has shown that glucosamine represses interleukin-1ß (Il1ß)-induced expression of MMP-3 in the SW-1353 chondrosarcoma cell line [10]. Interestingly, despite of numerous regulatory processes from MMP gene expression to the active enzyme, previous studies with colorectal and breast cancer specimens showed a close correlation between the level of gene expression and disease-free survival [11, 12].

Based on these studies, we hypothesized that (1) the reported effects of glucosamine are most probably not limited to the reported sarcoma cell types and may extend to osteosarcoma cells, (2) glucosamine suppresses the basal expression of MMPs in the absence of specific MMP inducers such as concanavalin A or PMA, and (3) glucosamine potentially suppresses MMP-3, MMP-9 and MMP-2 expression to different extents. Herein, we report, for the first time, that glucosamine sulfate exerts a pronounced suppressive effect on MMPs, particularly on MMP-3 and -9 in osteosarcoma cell lines in vitro.

## Methods

### Materials

All reagents were purchased from Sigma Aldrich (Sigma-Aldrich, St. Louis, USA) unless stated otherwise. Nutritional grade glucosamine sulfate powder (2(C_6_H_13_NO_5_)^.^H_2_SO_4_) was purchased from Vita Natura (Bonn, Germany). A glucosamine sulfate solution was prepared to a final concentration of 100 μg/ml in cell culture medium (detailed below). The solution was sterile filtered, aliquoted, and stored at −20 °C until further use.

### Cell culture

MG-63 osteosarcoma cells were obtained from the American Type Culture Collection (ATCC, Manassas, USA). SaOS-2 cells were purchased from Deutsche Sammlung für Mikroorganismen (DMSZ, Braunschweig, Germany). Both cell lines were cultured as a monolayer in Dulbecco’s Modified Eagle Medium (DMEM) supplemented with 1 % MEM-Vitamine, 1 % glutamine, 10 % fetal bovine serum (FBS), 1 U/mL penicillin/streptomycin and 2 % HEPES buffer (all obtained from Biochrom, Berlin, Germany) at 37 °C and 5 % CO_2_. Cells were passaged at 80–90 % confluency and culture medium was replaced every second day.

### Glucosamine sulfate treatment

Treatments were performed in a 6 well plates. MG-63 (2 × 10^6^) and SaOS-2 (4 × 10^6^) were seeded in 3 ml of medium and cultured for 24 h. Different seeding densities were used to compensate for the higher proliferation rate of MG-63 cells. The following day, the medium was replaced with fresh medium containing 2 % FBS and a final concentration of 10, 50 or 100 μg/ml glucosamine sulfate (achieved with a 1:9, 1:1 and 1:0 dilution of the glucosamine stock solution) and cells were incubated for 48 h. Following the incubation period, cells were dissociated with trypsin, counted and collected by centrifugation. RNA was then isolated from the cell pellets as described below.

### Toxicity assay

To detect potential toxicity associated with glucosamine sulfate treatment and exclude a downregulation of mRNA and protein expression due to cytotoxic cell death, a WST-1 cytotoxicity assay (Roche, Mannheim, Germany) was performed. Briefly, 2 × 10^5^ MG-63 and 4 × 10^5^ SaOS-2 were seeded in 200 μL medium in 48 well plates. Cells were then stimulated with different concentrations of glucosamine sulfate for 48 h (described above) prior to addition of 20 μl of WST reagent were added. Plates were incubated for 2 h before absorption was measured at 450 nm and a reference wavelength of 690 nm.

### Quantitative gene expression analysis

RNA was isolated from cells using the RNeasy Tissue Kit® (Qiagen, Hilden, Germany) according to the manufacturer’s instructions. In brief, MG-63 or SaOS-2 cells were resuspended in RLT buffer, transferred to a Qiashredder® and then lysed. RNA from lysates was immobilized on a silica matrix and eluted with distilled water. Harvested RNA was quantified by photometry.

Isolated RNA was transcribed to cDNA using QuantiTect® Reverse Transcriptions Kit (Qiagen, Hilden, Germany) according to the manufacturer’s instructions. The relative gene expression of MMP-2, -3 and -9 was quantified by real-time polymerase chain reaction (PCR) using a thermo cycler with TaqMan® Array and specific primers *(MMP-2: Hs01548727_m1; MMP-3: Hs01548727_m1; MMP-9: Hs01548727_m1;* glyceraldehyde 3-phosphate dehydrogenase *(GAPDH): NM_002046.3)* (Applied Biosystems, Grand Island, USA). GAPDH was used as internal control. The thermal cycler conditions were: 40 cycles consisting of a 15 s denaturation phase at 95 °C and a combined 1 min annealing and extension phase at 60 °C.

### Enzyme-linked immunosorbent assay (ELISA)

Protein analysis was performed from cells stimulated with 10 μg/ml glucosamine sulfate. After washing with PBS, a precipitation step with acetone and resuspension with cell extraction buffer (Invitrogen™, Frederick, USA) was performed.

Sandwich ELISA Kits for human MMP-3 and MMP-9 (Invitrogen™, Frederick, USA) were used for quantitative analysis according to the manufacturer’s instructions. In brief, 50 μl of diluted cell lysate was transferred to each microplate well previously coated with monoclonal antibody. After incubation with MMP-3 and MMP-9 specific detection antibodies as well as substrate solution the enzyme concentration was quantified spectrophotometrically in an ELISA Reader at 450 nm.

### Statistics

Quantitative PCR and ELISA data were compared using SPSS software (IBM, Armonk, USA) and the *t*-test for unpaired samples. The results are shown as mean ± standard deviation. A p-value of ≤0,05 was considered statistically significant.

## Results

**Fig. 1:**
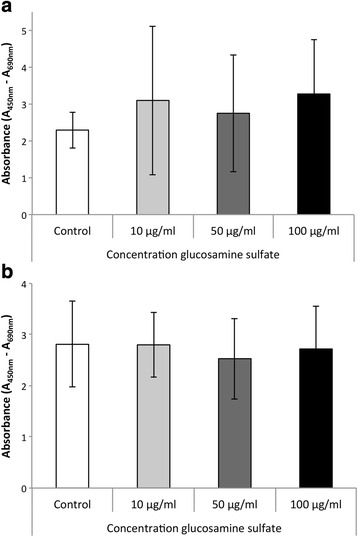
Cell viability as absorbance at A_450_–A_690_ in a WST-1 assay in the unstimulated control and upon administration of 10, 50 and 100 μg/ml glucosamine sulfate in MG-63 (**a**) and SaOS-2 cells (**b**); a statistical comparism was carried out for the control and each of the glucosamine sulfate treated groups; * indicates statistical significance with *p* ≤ 0,05

To assess the effect of glucosamine sulfate on MMP expression, glucosamine sulfate concentrations of 10 μg/ml, 50 μg/ml and 100 μg/ml were used. These concentrations were based on previous reports [8, 13]. Prior to quantitative analysis of MMP gene and protein expression, the potential cytotoxicity of the glucosamine sulfate treatment was examined. Glucosamine sulfate treatment did not significantly reduce the viability of either MG-63 (Fig. 1a) or Saos-2 (Fig. 1b) cells, indicating that the examined concentrations were not toxic.

**Fig. 2:**
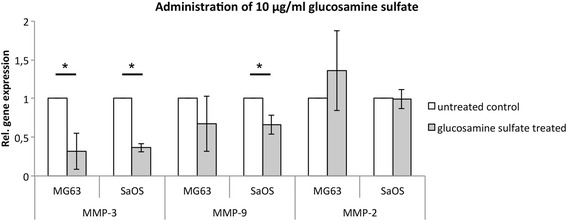
Relative gene expression of MMP-3, -9 and -2 in MG-63 and SaOS-2 cells upon administration of 10 μg/ml glucosamine sulfate; a statistical comparism was carried out for the control and each of the glucosamine sulfate treated groups; * indicates statistical significance with *p* ≤ 0,05

**Fig. 3:**
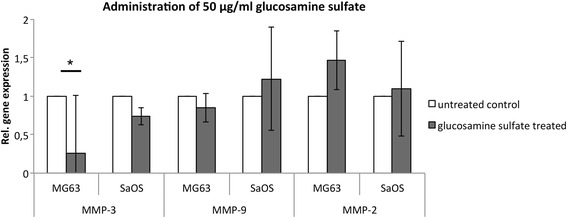
Relative gene expression of MMP-3, -9 and -2 in MG-63 and SaOS-2 cells upon administration of 50 μg/ml glucosamine sulfate; a statistical comparism was carried out for the control and each of the glucosamine sulfate treated groups; * indicates statistical significance with *p* ≤ 0,05

**Fig. 4:**
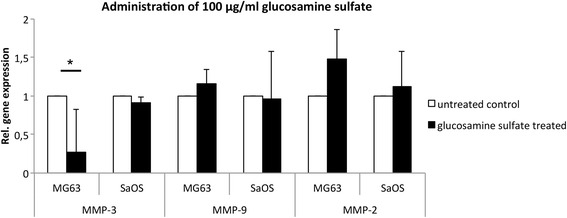
Relative gene expression of MMP-3, -9 and -2 in MG-63 and SaOS-2 cells upon administration of 100 μg/ml glucosamine sulfate; a statistical comparism was carried out for the control and each of the glucosamine sulfate treated groups; * indicates statistical significance with *p* ≤ 0,05

Subsequent analysis of MMP gene expression in response to 10 μg/ml glucosamine sulfate administration demonstrated a significant reduction of MMP-3 by approximately 68 % in MG-63 cells (*p* = 0,037) and 63 % in SaOS-2 cells (*p* = 0,003) compared to the untreated control (Fig. 2). Although MMP-9 expression was not significantly reduced in MG-63 cells, it was significantly reduced by approximately 34 % in SaOS-2 cells (*p* = 0,04; Fig. 2). Our analysis indicated that 10 μg/ml of glucosamine sulfate has no significant effect on MMP-2 gene expression in either MG-63 or Saos-2 cells (Fig. 2). Increasing the glucosamine sulfate concentration to 50 μg/ml significantly reduced MMP-3 gene expression by approximately 74 % in MG-63 cells (*p* = 0,021), while the reduction of MMP-3 in Saos-2 cells did not reach significance (Fig. 3). The expression of MMP-9 and MMP-2 was not significantly altered by 50 μg/ml glucosamine sulfate in either cell line (Fig. 3). Similar to the effect of 50 μg/ml glucosamine sulfate on MMP-3 expression in MG-63 cells, administration of 100 μg/ml resulted in a significant decrease of MMP-3 expression by about 73 % in MG-63 cells (*p* = 0,021; Fig. 4). However, the gene expressions of MMP-9 and MMP-2 were not significantly altered by 100 μg/ml glucosamine sulfate.

**Fig. 5:**
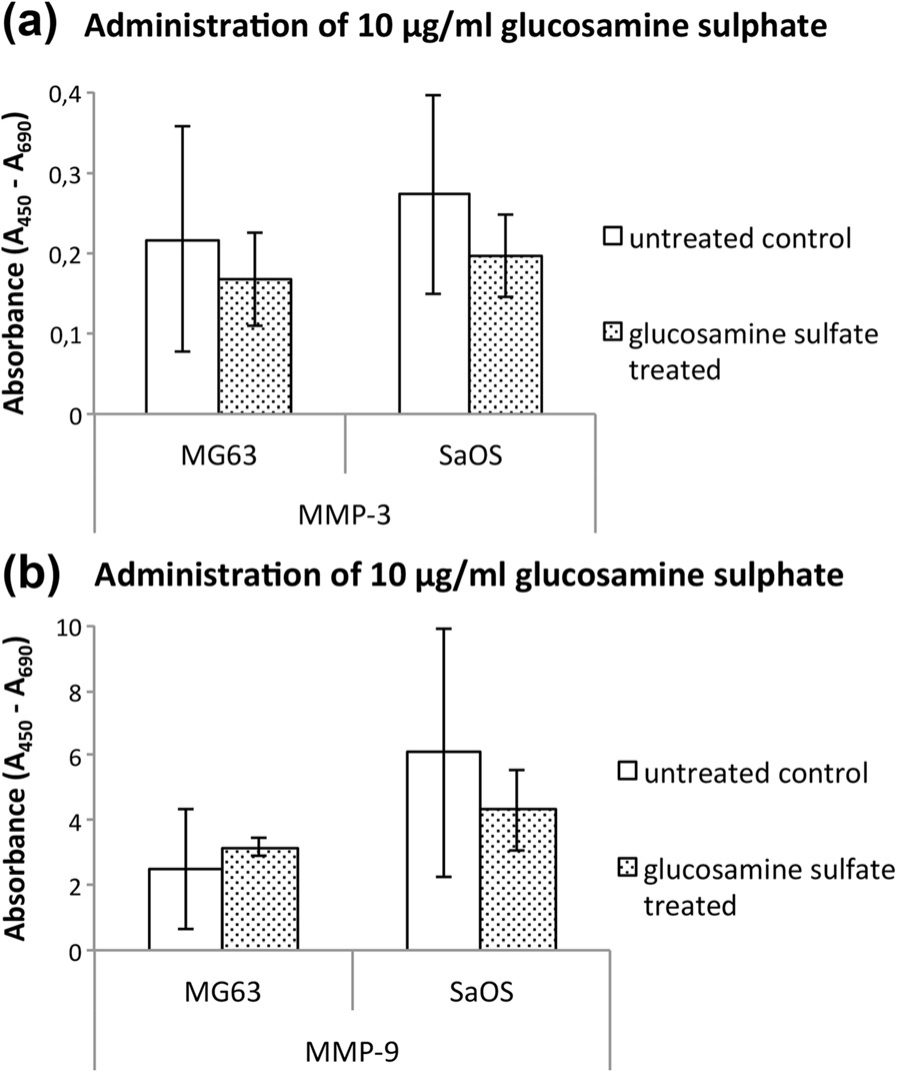
Protein levels as absorbance at A_450_–A_690_ in an ELISA assay of (**a**) MMP-3 and (**b**) MMP-9 in MG-63 and SaOS-2 cells upon administration of 10 μg/ml glucosamine sulfate; * indicates statistical significance with *p* ≤ 0,05

Protein expression analysis demonstrated similar results. Upon administration of 10 μg/ml glucosamine sulfate MMP-3 protein level was reduced by about 23 % in MG-63 (*p* = 0,60) and 28 % in SaOS-2 cells (*p* = 0,38) (Fig. 5a). MMP-9 revealed an insignificant increase of approximately 22 % upon stimulation with 10 μg/ml GlS in MG-63 cells (*p* = 0,54). SaOS-2 cells exhibited a decrease of MMP-9 protein level of about 29 % (*p* = 0,49) (Fig. 5b).

## Discussion

In this study, we observed that (1) glucosamine sulfate inhibits MMP gene and protein expressions in MG-63 and SaOS-2 osteosarcoma cell lines, (2) glucosamine sulfate suppresses basal MMP expression in osteosarcoma cells, and (3) glucosamine sulfate most significantly affects MMP-3 expression compared to MMP-9 and MMP-2.

MMPs are overexpressed in many different types of cancer, and consequently these proteinases are frequently investigated therapeutic target [14]. To date, a considerable number of pharmacological MMP inhibitors have been developed and tested in clinical trials [15]. However, their therapeutic efficacy thus far has proved limited [16]. The potential reasons for this lack of efficacy include: (1) limitations associated with the extensive homology between MMP catalytic domains; none of the tested drugs have been highly selective for specific MMPs, which could result in anti-tumor MMPs being inhibited; (2) unanticipated long-term drug intolerance that can reduce drug compliance; (3) the exclusion of early-stage cancer patients from clinical trials; these patients are anticipated to benefit most from MMP blockade; and (4) the drug doses used have been based on short-term kinetic studies in healthy volunteers and are not necessarily predictive of chronic therapeutic drug levels in cancer [16]. Thus, alternative strategies for MMP inhibition in cancer patients are of considerable interest.

In the present study, we demonstrate that a relatively low concentration of 10 μg/ml glucosamine sulfate reduces MMP-3 expression more than 50 % in both of the osteosarcoma cell lines investigated. This finding is particularly relevant because recent work by Tsai et al. found that MMP-3 is overexpressed in human osteosarcomas [17]. Further, their data identified miR-519d down-regulation via connective tissue growth factor (CTGF) signaling as a pathway osteosarcoma cells utilize to upregulate MMP-3 and MMP-2 expression [17]. But also mitogen-activated protein kinase (MAPK) pathways seem to play a pivotal role in mediating inflammatory and oncogenic signals and consequently inducing MMP-3 expression [18]. In this context Scotto d’Abuso and coworkers identified a decreased phosphorylation of c-jun-amino-terminal kinase (JNK) and p38 in human chondrocytes upon stimulation with glucosamine [19]. Moreover, their study showed a decreased activity of c-jun and junD depicting important transcription factors of MMPs [19]. The decreased phosphorylation of MAPKs by glucosamine may, in turn, be explained by coupling of N-acetylated glucosamine (N-acetylglucosamine) to serine or threonine residues of those proteins [19]. This O-glycosylation is thought to act in a manner analogous to phosphorylation [20, 21]. In support of these findings, recent clinical studies have demonstrated a close correlation between MMP-3 expression and the development of lung metastases in breast and lung cancers [22, 23]. In our study, which analized the expression of three MMPs, we found that glucosamine sulfate treatment exerted an unexpectedly specific effect. For example, the expression of MMP-3 was significantly reduced by glucosamine sulfate, but MMP-2 expression was not. In fact, a modest, yet statistically insignificant, increase was seen. Although these observations require further investigations, they suggest, that glucosamine sulfate is not a broad MMP inhibitor, but may rather be useful for more specific MMP suppression.

The data presented here support the potential use of glucosamine sulfate as a nutritional supplement in osteosarcoma patients for two main reasons. First, the glucosamine used in this study was a commercially available dietary glucosamine, formulated as glucosamine sulfate, which, together with glucosamine hydrochloride constitutes the principle nutritional formulation for glucosamine supplements [24, 25]. Previous studies have used either the non-dietary quaternized amino glucosamine [9] and N-acetyl-D-glucosamine [7], or sulfated glucosamine synthesized in-house [8]. Second, the most pronounced effect of glucosamine sulfate was observed at a concentration of 10 μg/ml. This concentration is within the range of the human serum concentrations that can be achieved with oral glucosamine intake, as previously published by Biggee et al. [26]. As such, it can be foreseen that glucosamine sulfate supplementation during osteosarcoma treatment could have a potentially beneficial effect. Upon diagnosis the vast majority of patients are treated with multi-agent neoadjuvant chemotherapy, followed by wide surgical resection of the tumor and adjuvant chemotherapy. Such regimes result in excellent primary tumor control, however, pulmonary metastases, which are challenging to control, develop in more than 30 % of patients [27]. One of the advantages of glucosamine of glucosamine dietary supplementation in osteosarcoma patients is that it will, most likely, integrate seamlessly into the standard treatment protocol. Other advantages of glucosamine dietary supplementation, namely the lack of toxicity, unrestricted availability, and low treatment costs this would enable supplementation to start permit upon initial diagnosis and continue over long periods of time, past primary tumor treatment. This may eventually reduce the incidence of pulmonary metastasis from osteosarcoma. As such, we anticipate that glucosamine sulfate treatment during osteosarcoma therapy will receive further attention, similar to the renewed interest in other nutraceuticals in augmenting standard cancer therapy [28]. However, we appreciate that the nutritional translation of the present findings will require a substantial further investigations in vitro and in preclinical models.

## Conclusion

In conclusion, we have shown that glucosamine sulfate exerts a pronounced suppressive effect on MMPs in osteosarcoma cell lines in vitro*;* this effect was most pronounced for MMP-3 and MMP-9 to a lesser extent. The data warrants further studies into the potential antitumor effect of glucosamine sulfate in osteosarcoma.

## Additional file

Additional file 1: Minimal data set underlying our findings. (XLSX 35 kb)

## Abbreviations

CTGF: Connective tissue growth factor
DMEM: Dulbecco’s Modified Eagle Medium
ELISA: Enzyme-linked immunosorbent assay
FBS: Fetal bovine serum
GAPDH: Glyceraldehyde 3-phosphate dehydrogenase
Il1ß: Interleukin-1ß
JNK: c-jun-amino-terminal kinase
MAPK: Mitogen-activated protein kinase
MMP: Matrix metalloproteinase
PCR: Polymerase chain reaction
PMA: Phorbol 12-myristate 13-acetate

## References

[1] Quastel JH, Cantero A (1953). Inhibition of tumour growth by D-glucosamine. Nature.

[2] Chesnokov V, Gong B, Sun C, Itakura K (2014). Anti-cancer activity of glucosamine through inhibition of N-linked glycosylation. Cancer Cell Int.

[3] Satia JA, Littman A, Slatore CG, Galanko JA, White E (2009). Associations of herbal and specialty supplements with lung and colorectal cancer risk in the VITamins and Lifestyle study. Cancer Epidemiol Biomarkers Prev.

[4] Bell GA, Kantor ED, Lampe JW, Shen DD, White E (2012). Use of glucosamine and chondroitin in relation to mortality. Eur J Epidemiol.

[5] Molnar Z, Bekesi JG (1972). Effects of D-glucosamine, D-mannosamine, and 2-deoxy-D-glucose on the ultrastructure of ascites tumor cells in vitro. Cancer Res.

[6] Molnar Z, Bekesi JG (1972). Cytotoxic effects of D-glucosamine on the ultrastructures of normal and neoplastic tissues in vivo. Cancer Res.

[7] Gervasi DC, Raz A, Dehem M, Yang M, Kurkinen M, Fridman R (1996). Carbohydrate-mediated regulation of matrix metalloproteinase-2 activation in normal human fibroblasts and fibrosarcoma cells. Biochem Biophys Res Commun.

[8] Rajapakse N, Mendis E, Kim MM, Kim SK (2007). Sulfated glucosamine inhibits MMP-2 and MMP-9 expressions in human fibrosarcoma cells. Bioorg Med Chem.

[9] Mendis E, Kim MM, Rajapakse N, Kim SK (2009). The inhibitory mechanism of a novel cationic glucosamine derivative against MMP-2 and MMP-9 expressions. Bioorg Med Chem Lett.

[10] Lin YC, Liang YC, Sheu MT, Lin YC, Hsieh MS, Chen TF, Chen CH (2008). Chondroprotective effects of glucosamine involving the p38 MAPK and Akt signaling pathways. Rheumatol Int.

[11] Zeng ZS, Huang Y, Cohen AM, Guillem JG (1996). Prediction of colorectal cancer relapse and survival via tissue RNA levels of matrix metalloproteinase-9. J Clin Oncol.

[12] McGowan PM, Duffy MJ (2008). Matrix metalloproteinase expression and outcome in patients with breast cancer: analysis of a published database. Ann Oncol.

[13] Zhang L, Liu WS, Han BQ, Peng YF, Wang DF (2006). Antitumor activities of D-glucosamine and its derivatives. J Zhejiang Univ Sci B.

[14] Deryugina EI, Quigley JP (2006). Matrix metalloproteinases and tumor metastasis. Cancer Metastasis Rev.

[15] Overall CM, Kleifeld O (2006). Tumour microenvironment - opinion: validating matrix metalloproteinases as drug targets and anti-targets for cancer therapy. Nat Rev Cancer.

[16] Zucker S, Cao J (2009). Selective matrix metalloproteinase (MMP) inhibitors in cancer therapy: ready for prime time?. Cancer Biol Ther.

[17] Tsai HC, Su HL, Huang CY, Fong YC, Hsu CJ, Tang CH (2014). CTGF increases matrix metalloproteinases expression and subsequently promotes tumor metastasis in human osteosarcoma through down-regulating miR-519d. Oncotarget.

[18] Chandhanayingyong C, Kim Y, Staples JR, Hahn C, Lee FY (2012). MAPK/ERK signaling in osteosarcomas, ewing sarcomas and chondrosarcomas: therapeutic implications and future directions. Sarcoma.

[19] Scotto d’Abusco A, Calamia V, Cicione C, Grigolo B, Politi L, Scandurra R (2007). Glucosamine affects intracellular signalling through inhibition of mitogen-activated protein kinase phosphorylation in human chondrocytes. Arthritis Res Ther.

[20] Zachara NE, Hart GW (2004). O-GlcNAc a sensor of cellular state: the role of nucleocytoplasmic glycosylation in modulating cellular function in response to nutrition and stress. Biochim Biophys Acta.

[21] Zachara NE, Hart GW (2006). Cell signaling, the essential role of O-GlcNAc!. Biochim Biophys Acta.

[22] Pettersson F, Del Rincon SV, Emond A, Huor B, Ngan E, Ng J, Dobocan MC, Siegel PM, Miller WH (2015). Genetic and pharmacologic inhibition of eIF4E reduces breast cancer cell migration, invasion, and metastasis. Cancer Res.

[23] Seccareccia E, Pinard M, Wang N, Li S, Burnier J, Dankort D, Brodt P (2014). The inhibitor of kappa B kinase-epsilon regulates MMP-3 expression levels and can promote lung metastasis. Oncogenesis.

[24] Navarro SL, White E, Kantor ED, Zhang Y, Rho J, Song X, Milne GL, Lampe PD, Lampe JW (2015). Randomized trial of glucosamine and chondroitin supplementation on inflammation and oxidative stress biomarkers and plasma proteomics profiles in healthy humans. PLoS One.

[25] Qato DM, Alexander GC, Conti RM, Johnson M, Schumm P, Lindau ST (2008). Use of prescription and over-the-counter medications and dietary supplements among older adults in the United States. JAMA.

[26] Biggee BA, Blinn CM, McAlindon TE, Nuite M, Silbert JE (2006). Low levels of human serum glucosamine after ingestion of glucosamine sulphate relative to capability for peripheral effectiveness. Ann Rheum Dis.

[27] Jaffe N (2009). Osteosarcoma: review of the past, impact on the future. The American experience. Cancer Treat Res.

[28] Ahmad A, Li Y, Sarkar FH (2016). The bounty of nature for changing the cancer landscape. Mol Nutr Food Res.

